# Shifting Focus: An Advocacy-Focused Analysis of Omega Beta Iota’s Membership and Political Engagement Strategies

**DOI:** 10.7759/cureus.105193

**Published:** 2026-03-13

**Authors:** Mishalle F Rashid, Syed A Rizvi

**Affiliations:** 1 Medicine, Edward Via College of Osteopathic Medicine, Blacksburg, USA; 2 Emergency Medicine, Louisiana State University Health Sciences Center, New Orleans, USA; 3 Internal Medicine, Louisiana State University Health Sciences Center, New Orleans, USA

**Keywords:** advocacy training, grassroots engagement, health policy, medical student leadership, membership development, omega beta iota, osteopathic advocacy, political engagement, professional development, student-run organizations

## Abstract

The National Osteopathic Political Advocacy Honor Society, Omega Beta Iota (OBI), wished to enhance the involvement of osteopathic medical students in the political advocacy aspects of American healthcare. During the COVID-19 pandemic, OBI’s main concern was retaining membership during turbulent times. However, with the return of inductee numbers to pre-pandemic levels, the 2023-2024 executive board aimed to shift focus from volume to quality, meaning purposeful engagement, leadership cultivation, and active involvement in advocacy-focused initiatives.

The primary focus of this advocacy analysis was to address the specific actions taken to adjust the value proposition of OBI to members. For example, OBI worked to increase value by expanding advocacy partnerships with groups such as the American Osteopathic Association (AOA) and American Association of Colleges of Osteopathic Medicine (AACOM), boosting its social media presence with consistent national-level updates, and creating clearer communication channels with chapter leaders. Development-centered approaches were also taken to address the active involvement gap between induction and sustained participation. Examples included enhanced alumni networking events, promoted as lifetime membership benefits; dedicated OBI presence at major osteopathic events, such as Doctors of Osteopathic Medicine Day (DO Day) and Osteopathic Medical Education Conference (OMED); and expanded opportunities for continued advocacy training. While short-term, these approaches were aimed at addressing long-term gaps through initiatives designed to strengthen advocacy training and member visibility.

## Editorial

Introduction

As the National Osteopathic Political Advocacy Honor Society, Omega Beta Iota (OBI) works to spotlight the vital contributions of osteopathic medical students to the U.S. political landscape. During the COVID-19 pandemic, the focus of OBI was primarily on maintaining continued interest within the society by sustaining membership induction metrics. However, in 2023-2024, as inductees returned to pre-pandemic numbers, OBI focused more on providing additional membership advantages and opportunities that allowed osteopathic medical students to distinguish themselves through leadership roles, grassroots activism, and participation in political events [1]. This aligned with recent findings that medical students are increasingly interested in advocacy roles beyond clinical care [2]. The purpose of this editorial is to examine how the 2023-2024 OBI Executive Board (E-Board) transitioned from prioritizing retention to emphasizing value in membership benefits through these activities.

Before the pandemic, inductee numbers consistently exceeded 200 per year; during the pandemic, they dropped by nearly half, reaching lows of around 110-120 inductees annually. By 2023-2024, however, improved recruitment and programmatic changes resulted in membership rebounding to 230 inductees, surpassing pre-pandemic levels. These numbers provided the foundation for a pivot in strategy.

New educational programs to meet student expectations

The strategic approach established at the beginning of the 2023-2024 OBI E-Board tenure aimed to better market current offerings and develop new opportunities throughout the year. From a marketing standpoint, OBI developed stronger relationships with other osteopathic advocacy partners, such as the AOA, AACOM, and the Student Osteopathic Medical Association (SOMA), by establishing more presence on social media, partnering in joint advocacy campaigns, and creating collaborative webinars with affiliate leaders. One-on-one opportunities with advocacy partners were expanded via leadership roundtables, and member newsletters helped keep students and affiliate organizations informed.

In terms of development, the E-Board observed that, though many students were inducted into membership, there existed a desire for clearer lines of progression toward leadership, formalized training in advocacy, and national-level exposure. In response to these demands, the E-Board established and implemented effective educational programming centered on short-term and long-term solutions. These types of interventions have been found to improve political and social awareness in medical education learners [3]. Short-term solutions consisted of greater emphasis on the lifetime membership component, greater exposure to advocacy leaders through networking partnerships with alumni, as well as greater OBI exposure at major osteopathic conferences. Long-term solutions consisted of the implementation of the National Advocacy Training Series (a tiered workshop featuring affiliate speakers over multiple sessions) as well as the OBI Policy Leadership Program (a year-long mentorship program in which students would be paired with advocacy professionals).

New programs lead to increased applications and inductees

By applying this approach, OBI saw record-breaking numbers of both applications and inductees. Applications rose by 35% compared to the previous year, and inductee numbers (230) exceeded pre-pandemic averages (200). This increase in numbers allowed for increased presence within affiliate organizations, successful implementation of long-term programs, and the creation of new partnerships to develop more opportunities for members. The new programs also increased resident involvement on the E-Board by expanding the number of available leadership positions and allowing members to actively participate within the organization rather than serving passively as honorary members.

Expanding engagement through value-driven membership

By undergoing a strategic shift from focusing on membership volume to membership value within OBI, there was a marked increase in engagement and organizational growth. Engagement was measured by the number of students participating in alumni networking events, national conferences, and advocacy trainings. Attendance at these activities grew from fewer than 50 students in 2021-2022 to over 150 in 2023-2024. By implementing targeted marketing campaigns and developing meaningful engagement opportunities, the organization both enhanced the caliber of its applicants and expanded its collaborative footprint across affiliated advocacy coalitions. Evidently, students demonstrated a greater interest in organizations with active participation and leadership opportunities beyond medical school. The increased level of engagement and contribution of E-Board members reflected continuous advocacy and OBI’s capacity to provide longitudinal mentorship. It also reflected the effectiveness of methods that promoted engagement beyond the preclinical years of medical school.

Advocacy as a core professional responsibility

As a clear takeaway, organizational membership was more effective when membership was not regarded merely as an honor, but as a means to grow personally and professionally, and as a gateway to an organized initiative that honed advocacy and political engagement [4]. This produced more committed, effective, and politically active future physicians. Advocacy was recognized not only by researchers but also by major medical organizations, such as the AOA, AACOM, and the American Medical Association (AMA), as a core professional responsibility within modern medical training [5]. The integration of policy within healthcare education further depicted the need to educate future physician leaders in these areas. OBI's model provides a template for similar advocacy-based student organizations to create lasting value through their programming. By sharing the details of program design, membership data, and engagement outcomes, this editorial aims to provide other groups with practical strategies for transforming their own organizations.

Conclusion

In introducing our advocacy objective, we established that qualitatively focused membership upgrades, rather than volume-based membership, generated greater organizational interest and provided more solid groundwork for the success of advocacy efforts. This change of focus ensured that members not only felt appreciated but also became actively engaged in advocacy work. The presence of the alumni network, organized through regular networking events and mentorship initiatives, as well as increased OBI presence at the national level, reinforced the need for long-term dedication to policy engagement. Leadership development pathways were also established, allowing progression from chapter-level officers to the National E-Board.

Furthermore, the newly established development and leadership training pipelines served as sustainable models for other student-led civic engagement organizations. Consistent with previous research, this work showed that purposeful membership and meaningful engagement, especially among medical students, amplified the impact of their advocacy work. As legislative decision-making continues to involve healthcare, these advocacy skills remain vital for the professional development of future physicians. The training they received during medical school profoundly shaped the socially responsible and socially engaged physicians of the future. This paper provides a framework for other organizations interested in expansion, demonstrating that focusing on quality engagement rather than volume creates sustainable growth and long-lasting impact.
